# Infrared diode laser enhances human periodontal ligament stem cells behaviour on titanium dental implants

**DOI:** 10.1038/s41598-024-54585-w

**Published:** 2024-02-20

**Authors:** Mohamed M. Abo El-Dahab, Ghada Nour El Deen, Riham M. Aly, Mostafa Gheith

**Affiliations:** 1 Department of Basic Dental Science, Oral and Dental Research Institute, National Research Centre, 33 El Buhouth St., Dokki, Cairo 12622 Egypt; 2 Stem Cell Laboratory, Center of Excellence for Advanced Sciences, National Research Centre, Cairo, Egypt; 3 Molecular Genetics and Enzymology Department, Human Genetic and Genome Research Institute, National Research Centre, Dokki, Cairo Egypt; 4 National Institute of Laser Enhanced Sciences, Cairo University, Giza, Egypt

**Keywords:** Periodontal ligament stem cells, Infrared diode laser, Osteogenic differentiation, Dental implants, Attachment, Dentistry, Stem-cell research, Cell biology, Medical research

## Abstract

Low level laser treatment (LLLT) is known for its photobiostimulatory and photobiomodulatory characteristics, which stimulate cell proliferation, increase cellular metabolism, and improve cellular regeneration. The objective of the present research was to assess the possible influence of infrared diode laser irradiation on the behaviour, attachment, and osteogenic differentiation of human periodontal ligament stem cells (hPDLSCs) seeded on different types of dental implants. Two distinct types of implants, one subjected to laser surface treatment and the other treated with acid etching, were longitudinally divided into two halves and submerged in six wells culture plates. Both implants were subjected to infrared diode laser treatment, and subsequently, the morphology and attachment of cells were examined using scanning electron microscopy (SEM) after 14 and 21 days. The behaviour of (hPDLSCs) towards two types of implants, when exposed to osteogenic medium and low-level laser therapy (LLLT), was assessed using quantitative real-time polymerase chain reaction to measure the expression of stemness markers and osteogenic markers. The scanning electron microscopy (SEM) demonstrated that the application of infrared diode laser irradiation substantially improved the attachment of cells to both types of implants. The stemness gene markers were significantly down regulated in cells seeded on both surfaces when challenged with osteogenic media in relation to control. At 14 days, early osteogenic markers*,* were upregulated, while late osteogenic markers, were downregulated in both challenged groups. At the 21-day mark, hPDLSCs seeded on an acid-etched implant exhibited increased expression of all osteogenic markers in response to stimulation with osteogenic media and infra-red diode laser, in contrast to hPDLSCs seeded on a laser surface treated implant under the same conditions. Finally, the findings of our research revealed that when subjected to infrared diode laser, human periodontal ligament stem cells cultured on both types of implants demonstrated improved cellular attachment and differentiation. This suggested that infrared diode laser enhanced the activity of the cells surrounding the implants. Hence, the use of infrared diode laser could be pivotal in improving and expediting the clinical osseointegration process around dental implants.

## Introduction

Laser technology has been employed in the field of dentistry for more than thirty years to address a wide range of oral disorders and diseases. Laser techniques provide a more convenient treatment alternative for various dental treatments for both hard and soft tissues, in contrast to conventional dental treatments^1,2^. Table 1 provides a comprehensive overview of the different types of lasers utilized in dentistry, highlighting their distinct applications, power levels, and wavelengths. Among the various dental applications of lasers is implant removal^3^. This approach permits precision cutting of both soft and hard tissues and is non-invasive. Moreover, laser application allows cutting of the peri-implant bone selectively and with less invasiveness than alternative techniques. Although lasers have demonstrated efficacy in numerous dental procedures, their utilization is subject to potential contraindications and limitations^4^. Hard lasers have the potential to harm the dental pulp, and certain laser operations may still necessitate the use of anaesthesia.

**Table 1:** Types of lasers used in dentistry.

Type	Application	Wavelength	Dosage
Diode lasers	Used for soft tissue procedures, such as gum reshaping and periodontal treatment	‘Low level or non-surgical diode lasers’ (630–670, 800–830, and 900–940 nm) Surgical diode lasers” (445–455, 800–830, 940, and 970–980 nm)	Lower exposure parameters (2–4 J/cm^2^) are used for bio stimulation, and higher exposure parameters (10–12 J/cm^2^) for analgesia and desensitisation procedures
Nd:YAG lasers (neodymium yttrium aluminium garnet)	Used for procedures like root canal sterilization and periodontal treatment	1064 nm	Parameters can vary depending on the specific dental application and individual patient factors
Erbium lasers	Used for both hard and soft tissue procedures, including bone shaping and removing dental caries	2940 nm	Er:YAG laser pulses from 15 to 20 pulses per second can be used to induce analgesia in hard and soft tissues, allowing cutting of teeth, bone, and soft tissues with little or no local anaesthesia
CO_2_ lasers	Used for soft tissue procedures and have applications in oral surgery	9600 nm, 10,600 nm	Parameters can vary depending on the specific dental application and individual patient factors

In dentistry, diode lasers are regarded as safer than other laser sources because they are utilized exclusively at an extremely close distance, preventing injury from "beam escape"^1^. Clinical evidence indicates that diode lasers provide a number of benefits in comparison to conventional surgical procedures, such as enhanced precision and area sterilization. Diode lasers are economical, portable, and practical^2^. They are more suitable for use in close proximity to implants than electrosurgery because they produce less heat and do not conduct electricity. By means of fibers with diameters of 600, 400µ, and 200µ, the laser energy is capable of penetrating deep periodontal fissures and imparting its therapeutic effects. Additionally, low-level laser therapy (LLLT) induces beneficial outcomes in terms of fibroblast production, collagen synthesis, and neurotransmitter levels^5^. LLLT is a therapeutic technique that uses low-intensity laser light to stimulate cellular processes and enhance the healing of tissues. It orchestrates the process of tissue repair through stimulating cellular proliferation and differentiation in addition to modulating the secretion of essential cytokines and growth factors necessary for angiogenesis and regeneration^6^.

In context of regeneration, stem cells are regarded as pivotal components. Considerable research has been directed to the investigation of dental-derived stem cells owing to their remarkable abilities in terms of self-renewal, differentiation, and proliferation^7–11^. PDLSCs, which are a type of dental derived stem cells, are known for their ability to differentiate into multiple cell types and their enhanced ability to grow and divide^12^.

In the field of implantology, the success of dental implants hinges on the osseointegration of the implant with the adjacent alveolar bone and periodontal ligaments. This process is essential for ensuring the stability and long-lasting durability of the implant^13^. To achieve successful integration of the implant with the surrounding bone, a series of cellular processes takes place, leading to bone remodeling. However, it is important to note that both the material and microstructure of the implant play a critical role in this process, as it appears to influence the rearrangement of bone cells at the interface between the bone and the implant^14–17^.

Titanium is the most commonly utilized material in dental implants mainly due to its superior biocompatibility properties. Moreover, the structural design and surface attributes of titanium dental implants exert a significant impact on the proliferation and differentiation of cells. Several clinical reports have documented improved success rates of titanium dental implants^18,19^. However, ongoing efforts have been undertaken to propose various approaches that aim to optimize the process of osseointegration and speed its completion. Enhancing the surface topography of titanium dental implants may improve cellular adhesion and differentiation. Implant surface roughening and various coating processes are well-established and widely acknowledged technology^20–23^. Chemical or physical modifications of titanium implant surfaces, can also enhance cell adhesion and differentiation^24^. Additionally, laser texturing of implant surface is currently recognized as a highly effective method for minimizing adherence of microorganisms to implant surfaces in an attempt to avoid peri-implantitis^3^.

It was recently reported that by modifying the roughness and porosity of the implant surface, LLLT and diode laser contribute to enhancing osseointegration and accelerating bone formation around dental implants^5,25^. Additionally, the combination of acid etching and laser treatment has been suggested as a potential alternative approach to surface modification of dental implants^26^. This combination has demonstrated improved osseointegration and accelerated bone regeneration in proximity of dental implants. Combining periodontal ligament stem cells (PLSCs) with dental implants has demonstrated comparable results in improving the osseointegration process and promoting the regeneration of periodontal ligaments^13,27^.

We hypothesized that the combination of hPDLSCs with low-level laser therapy may improve osseointegration and cellular attachment, thereby enhancing the performance of dental implants^13^. Thus, the aim of the current study was to evaluate the in vitro effect of infrared diode laser application on the behavior of human periodontal ligament stem cells seeded on different dental implant surfaces in terms of proliferation, attachment, and osteogenic differentiation potential. Determining the potential impact of infrared diode laser on human periodontal ligament stem cells cultured on dental implants constituted the primary outcome. While identifying the optimum implant type that responded effectively to this combination in terms of cellular attachment and osteogenic differentiation constituted the secondary objective.

## Results

### Isolation of hPDLSCs

**Figure 1:**
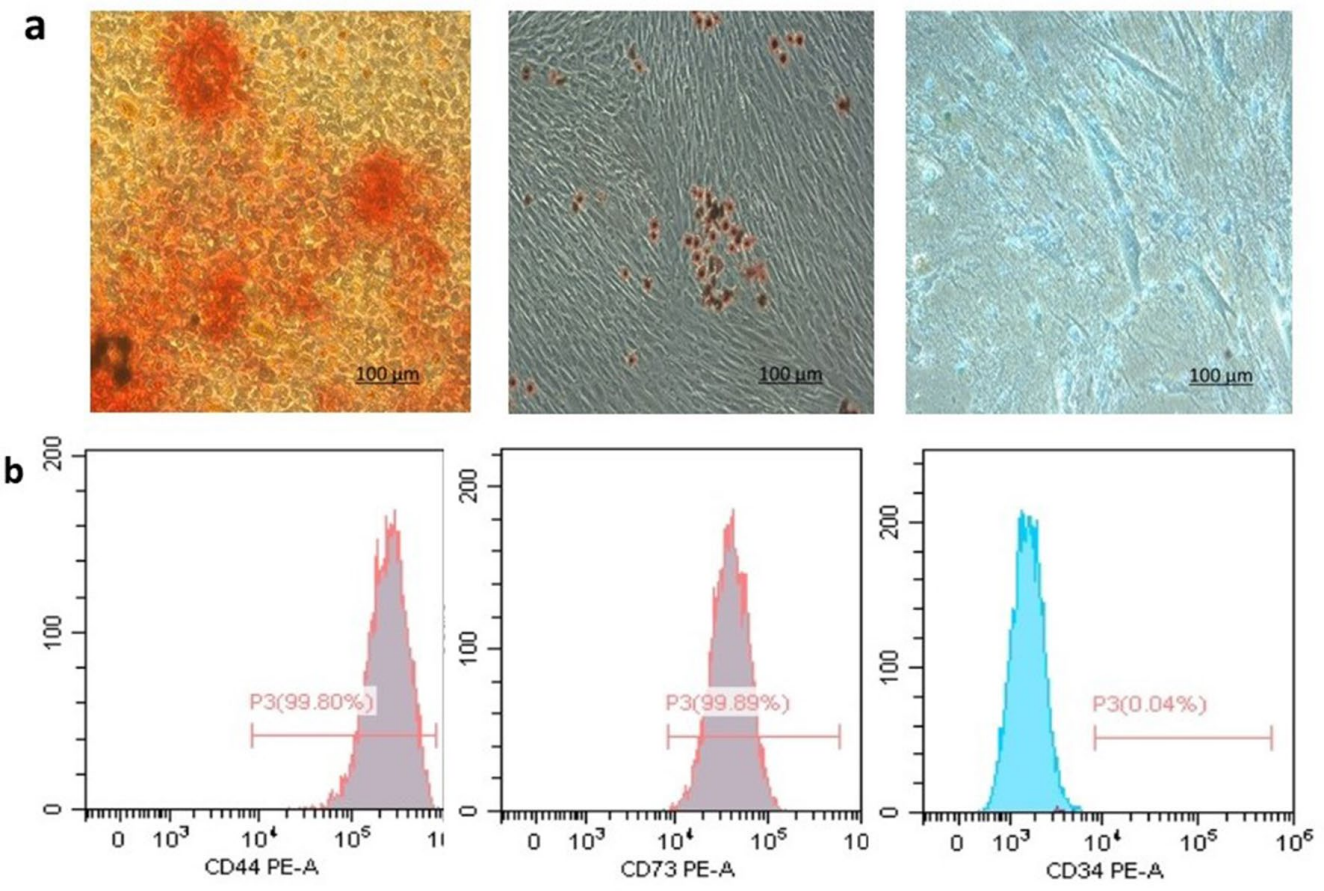
Characterization of DPSCs by multi-lineage differentiation and flow cytometry. (**a**) Multilineage differentiation potential. Isolated DPSCs illustrated positive Alizarin Red Stain indicative of osteogenic differentiation (left), positive adipogenic differentiation as indicated by Oil Red Stain (middle) and finally chondrogenic differentiation was positively expressed by Alicain blue stain (right). FACs analysis (**b**) indicated positive expression of mesenchymal stem cell markers CD44 and CD 73 and negative expression of haematopoietic marker CD 34.

**Figure 2:**
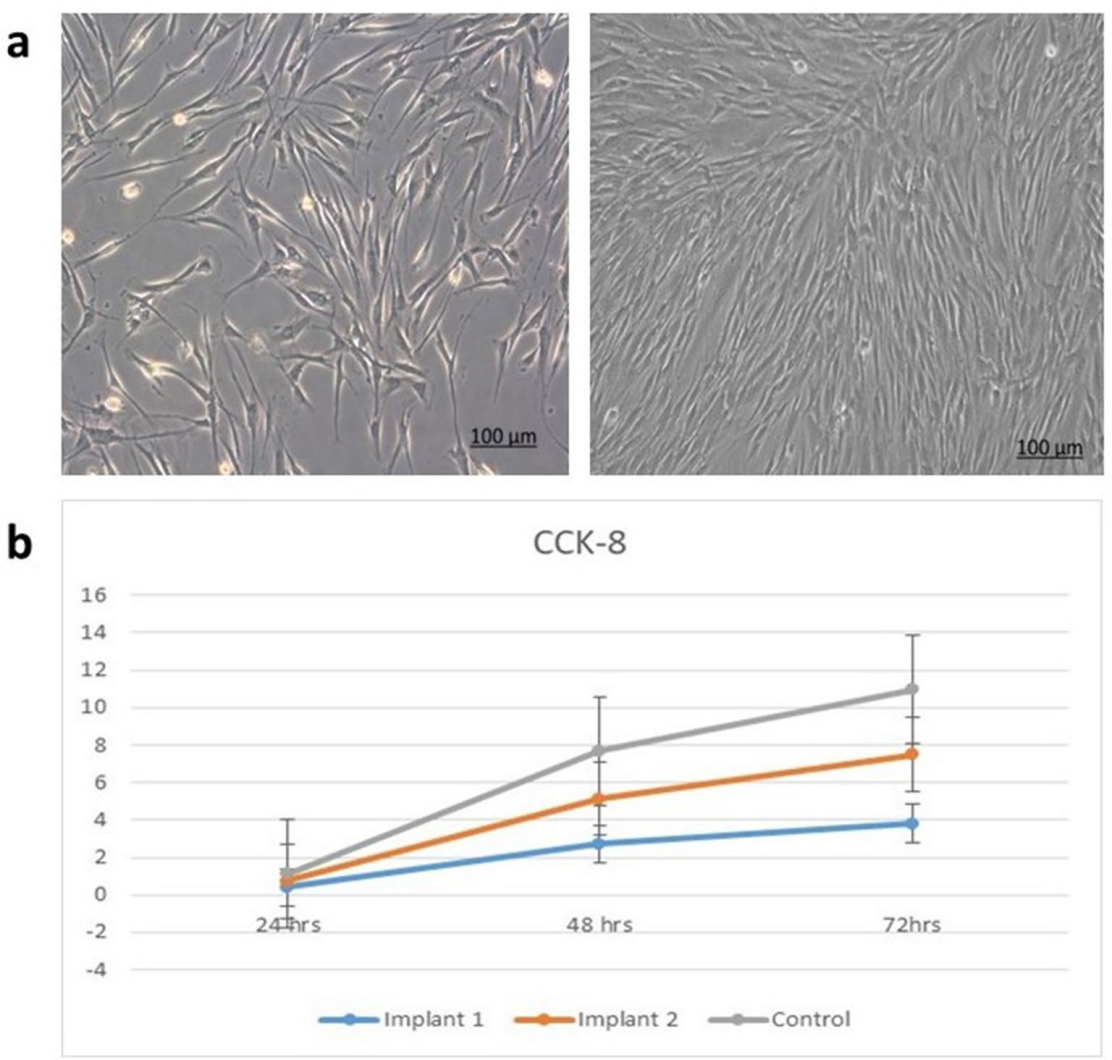
Photomicrograph illustrating hPDLSCs culture at various phases (**a**) single cell attachment five days after isolation (left), cells reaching confluence (right) Magnification ×100. (**b**) Graph presenting the proliferation curve of hPDLSCs seeded on implant surfaces following irradiation after 24, 48 and 72 h. Bars represent standard error. (Implant1 = laser treated), (Implant 2 = acid etched).

Following the third day of culture single spindle shaped started to attach to the culture plate. These cells exhibited round nuclei. After the third passage, the cells started to increase both in size and in number and exhibited radial growth pattern (Fig. 2a). The flow cytometry results (Fig. 1) demonstrated negative expression of CD35 (0.04%) and positive expression of CD44 (99.8%) and CD73 (99.89%). These results indicated that mesenchymal identity of the isolated cells. This was also confirmed by successful multilineage differentiation potential of the isolated cells into osteogenic, adipogenic and chondrogenic lineage (Fig. 1).

### CCK‑8 proliferation test results

The proliferation rate of irradiated hPDLSCs seeded on both types of implants was monitored and a proliferation curve was plotted. After 48 h, the proliferation rate increased, and this increase continued after 72 h in all groups (Fig. 2b). hPDLSCs seeded on acid etched implants demonstrated slightly higher rate of proliferation, however there was no significant significance between both types of implants.

### Observation of PDLSCs attachment and adhesion on the surface of the titanium disk using scanning electron microscope (SEM)

**Figure 3:**
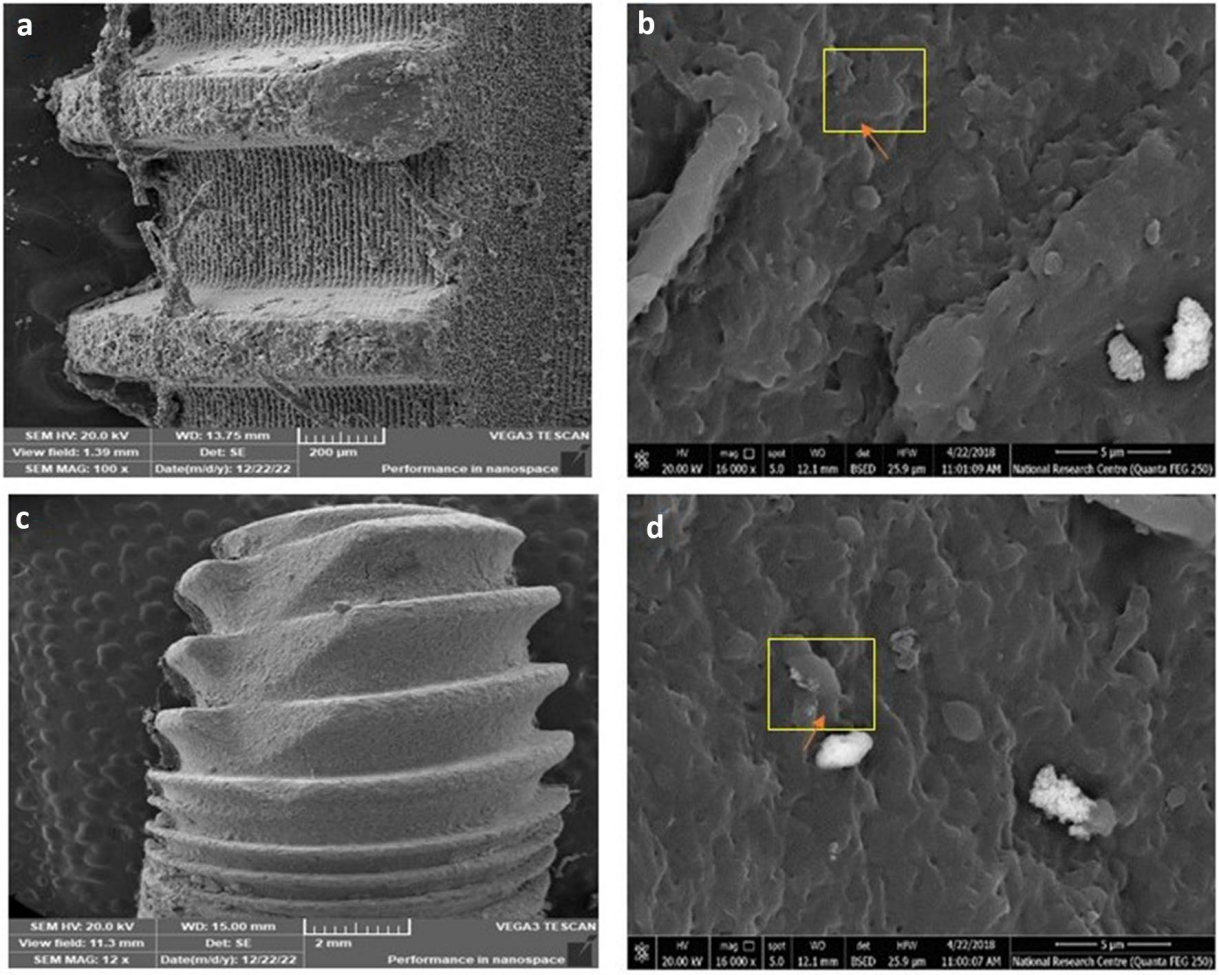
Scanning electron microscopy (SEM) illustrating the attachment of hPDLSCs on laser treated implants (**a**) and acid etched implants (**c**) before induction of osteogenic differentiation. Cells illustrated typical mesenchymal stem cells’ appearance (**b**, **d**) on both types of implants where the cell size was large (yellow box), stellate in shape and rich in filopodia (arrow) (magnification: (**a**) ×100, (**b**) ×16, (**c**) ×12, (**d**) ×16).

**Figure 4:**
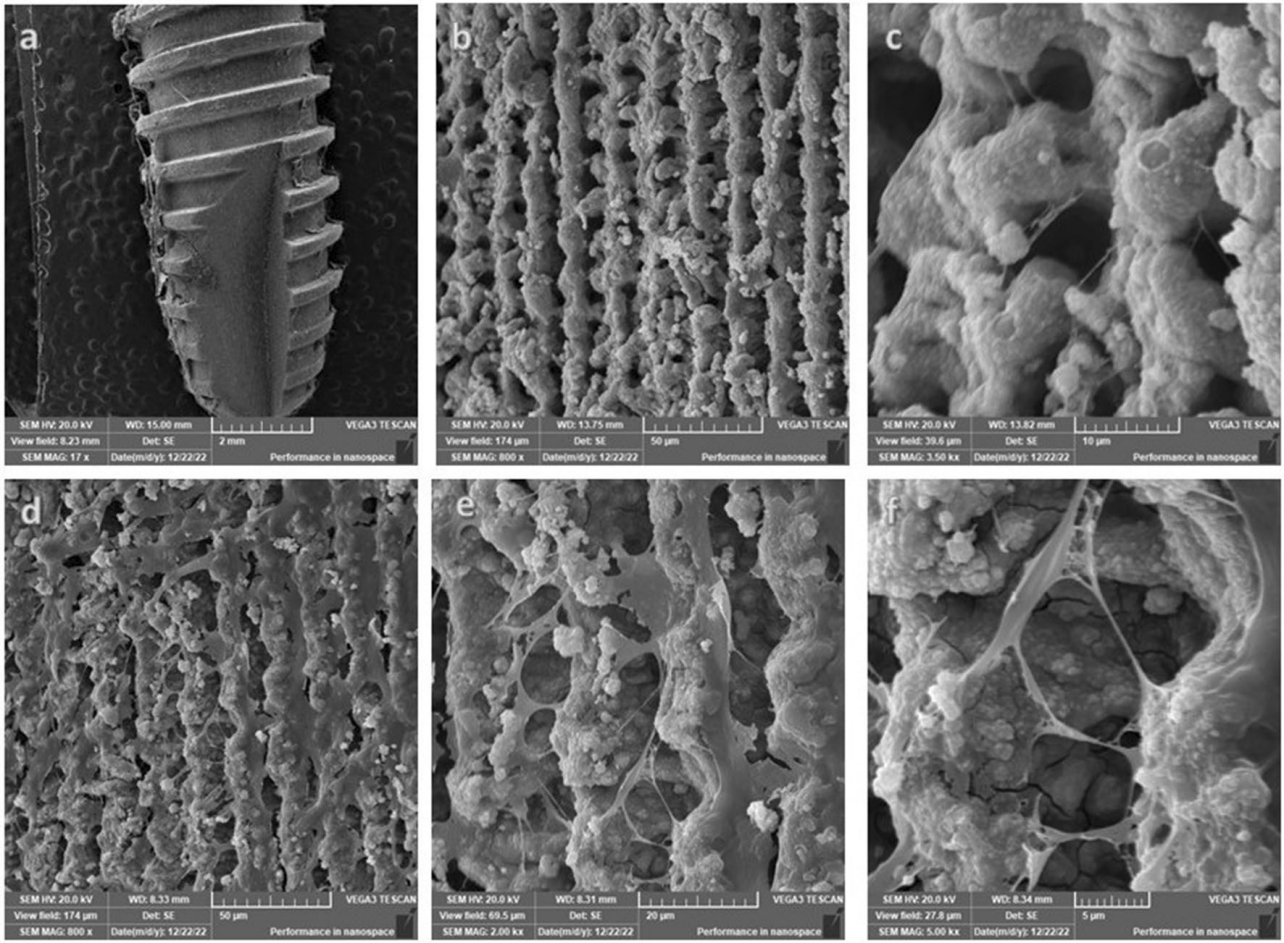
SEM observation of hPDLSCs seeded over laser treated dental implants. First row after 14 days of differentiation and second row after 21 days of differentiation. The cell bodies were full, and the cells were connected via synaptic extension. The cells exhibited intercellular connections through pseudopodia which projected out of the cell body attached to the surface of the implant. Filopodia were long and thin in appearance (**f**) (magnification: **a** ×17, **b** ×8, **c** ×35K, **d** ×800, **e** ×2, **f** ×5).

**Figure 5:**
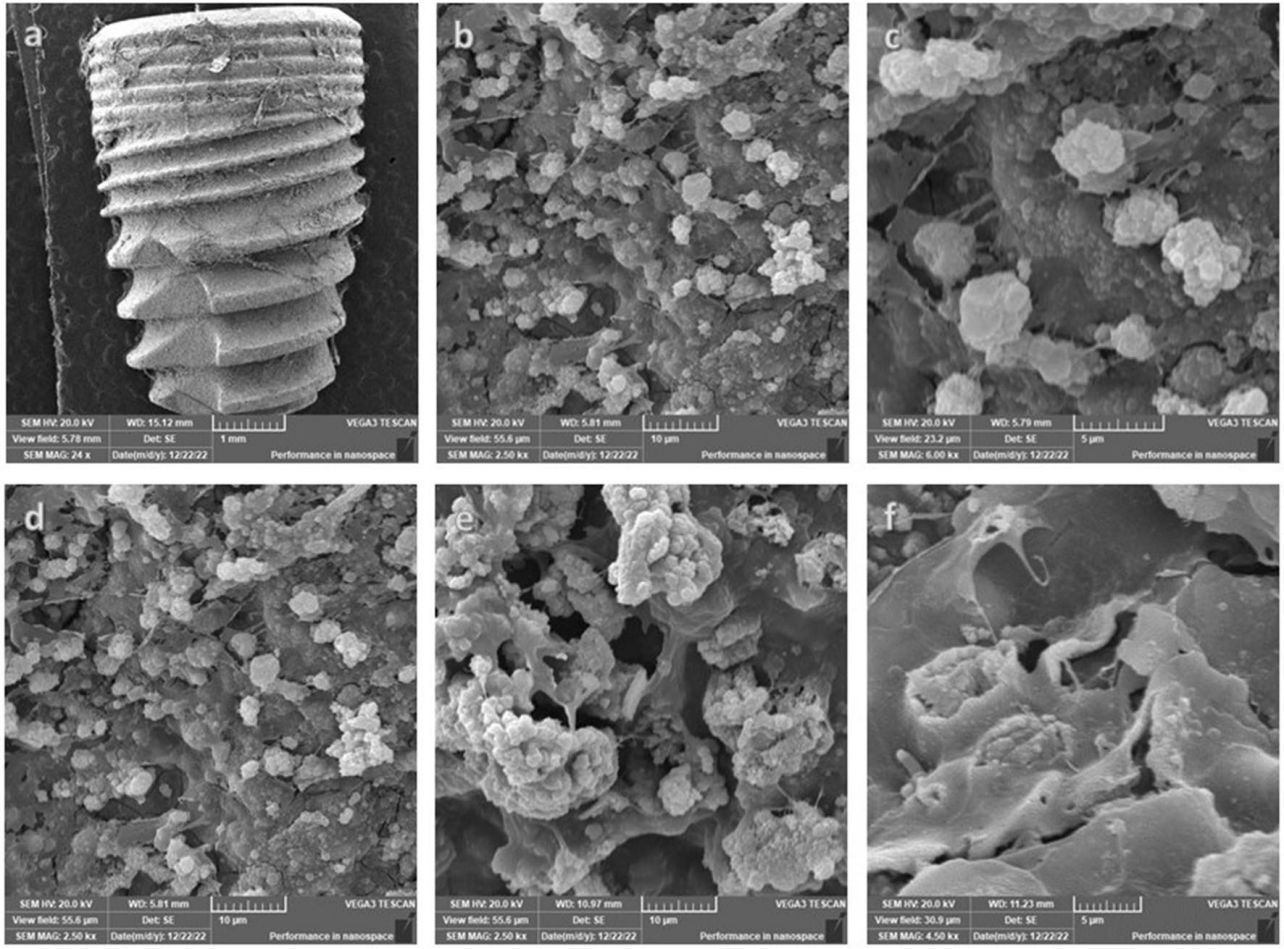
SEM observation of hPDLSCs seeded over acid etched dental implants. First row after 14 days of differentiation and second row after 21 days of differentiation. Cell bodies illustrated a fuller and rounder appearance and were connected via synaptic pseudopodia. The number of cells increased after 21 days (**d**) with more pronounced filopodia which are short and thick in appearance (**e,f**) (magnification: (**a**) ×24, (**b**) ×250, (**c**) ×6K, (**d**) ×2.5K; (**e**) ×2.5K, (**f**) ×4K).

SEM was used to observe the morphology of irradiated hPDLSCs seeded over both types of implants. Figure 3 demonstrates hPDLSCs cellular attachment on both implant surfaces prior to osteogenic differentiation. At this stage, the cells illustrated typical mesenchymal stem cells’ appearance where the cell size was large and rich in filopodia indicating growth and biocompatibility. Following the induction of osteogenic differentiation, the cell bodies became fuller, and the cells were connected via synaptic extension (Figs. 4, 5). The cells exhibited intercellular connections through pseudopodia, those pseudopodia projected out of the cell body and were attached to the surface of the implants. However, hPDLSCs illustrated a rather more stretched with more filopodia on laser treated implants compared to acid etched implants. Moreover, an organised channelled appearance of hPDLSCs attachment on the laser treated implants was very obvious (Fig. 4b) as opposed to the random growth and attachment of cells on the acid treated implants (Fig. 5b). This pattern was more evident after 3 weeks of differentiation (Figs. 4d–f and 5d–f). However, there was a more apparent increase in hPDLSCs attached to the acid etched implants compared to the laser treated implants. hPDLSCs attached to laser treated implants demonstrated fewer number of cells with less prominent filopodia which were long and thin in appearance (Fig. 4e, f). Signs of calcification were evident on both types of implants with a more pronounced appearance on acid etched implants especially after 3 weeks (Fig. 5d, e).

### Surface chemistry analysis

**Figure 6:**
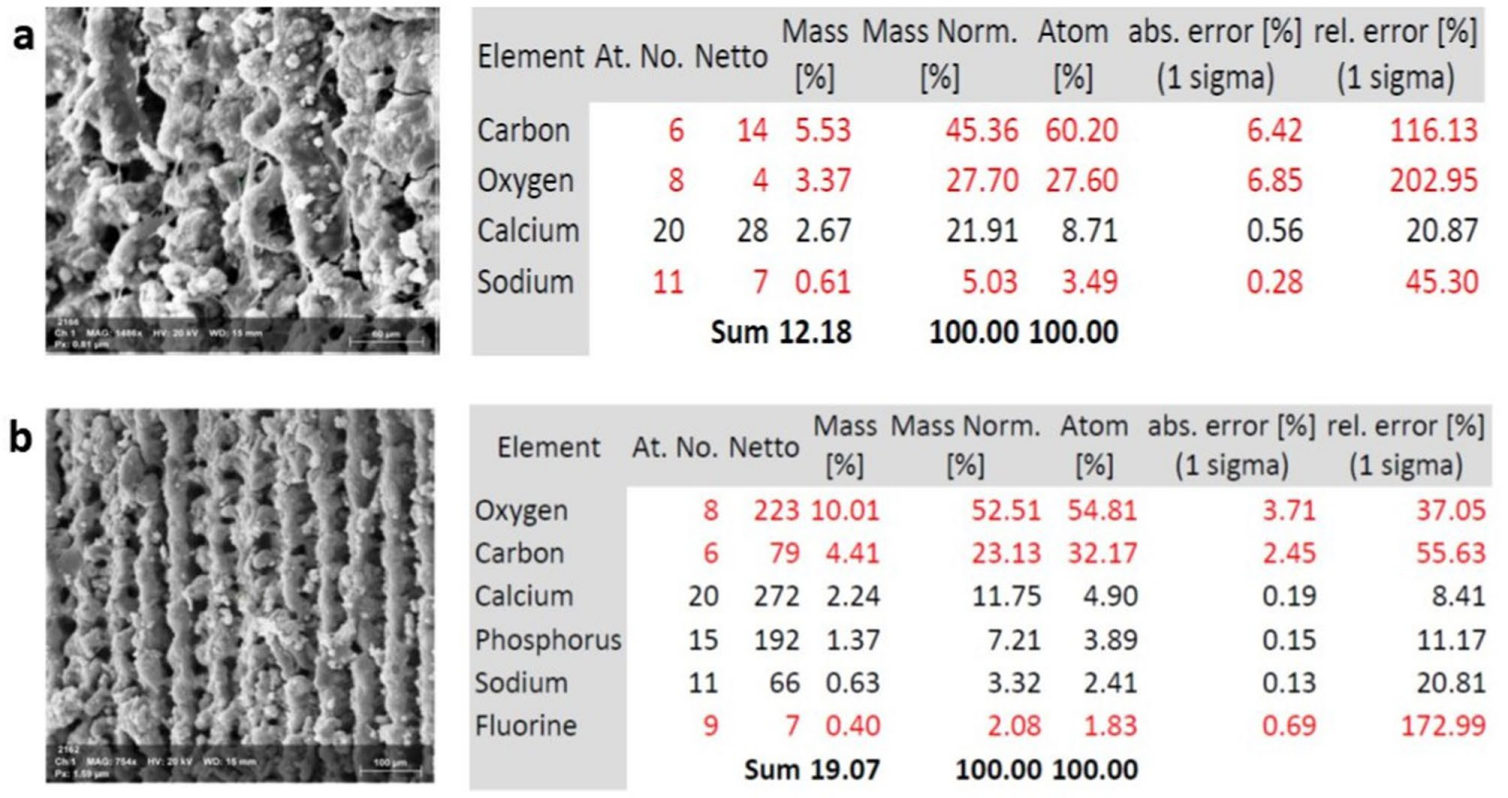
Scanning electron microscopy images of hPDLSCs after induction of differentiation on laser treated implants. After 14 days (**a**) and after 21 days (**b**) at magnification ×800 (left) and EDX analysis (right).

**Figure 7:**
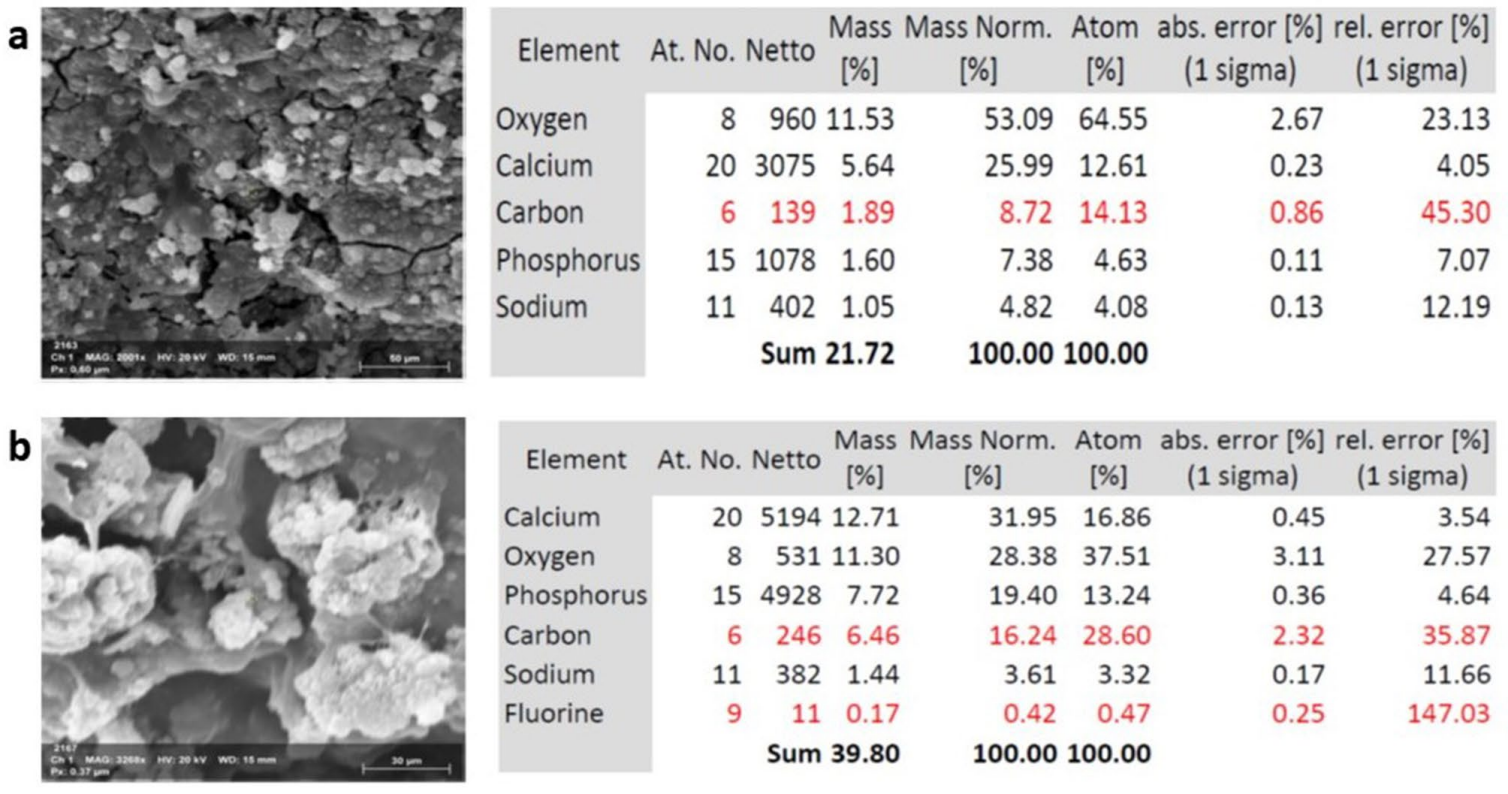
Scanning electron microscopy images of hPDLSCs after induction of differentiation on acid etched implants. After 14 days (**a**) and after 21 days (**b**) at magnification ×800 (left) and EDX analysis (right).

The chemical surfaces of both types of implants were assessed with SEM–EDX (Figs. 6, 7). According to the analysis diagram, O, and C, Ca, P and Na elements were detected on all implant surfaces after 14 days of culture in osteogenic media. After 21 days, P, and FL, were detected in very small amounts, however P was detected in acid etched implants as early as 14 days.

### Real-time polymerase chain reaction (PCR) analysis of adhesion- and osteogenesis-related gene expression

**Figure 8:**
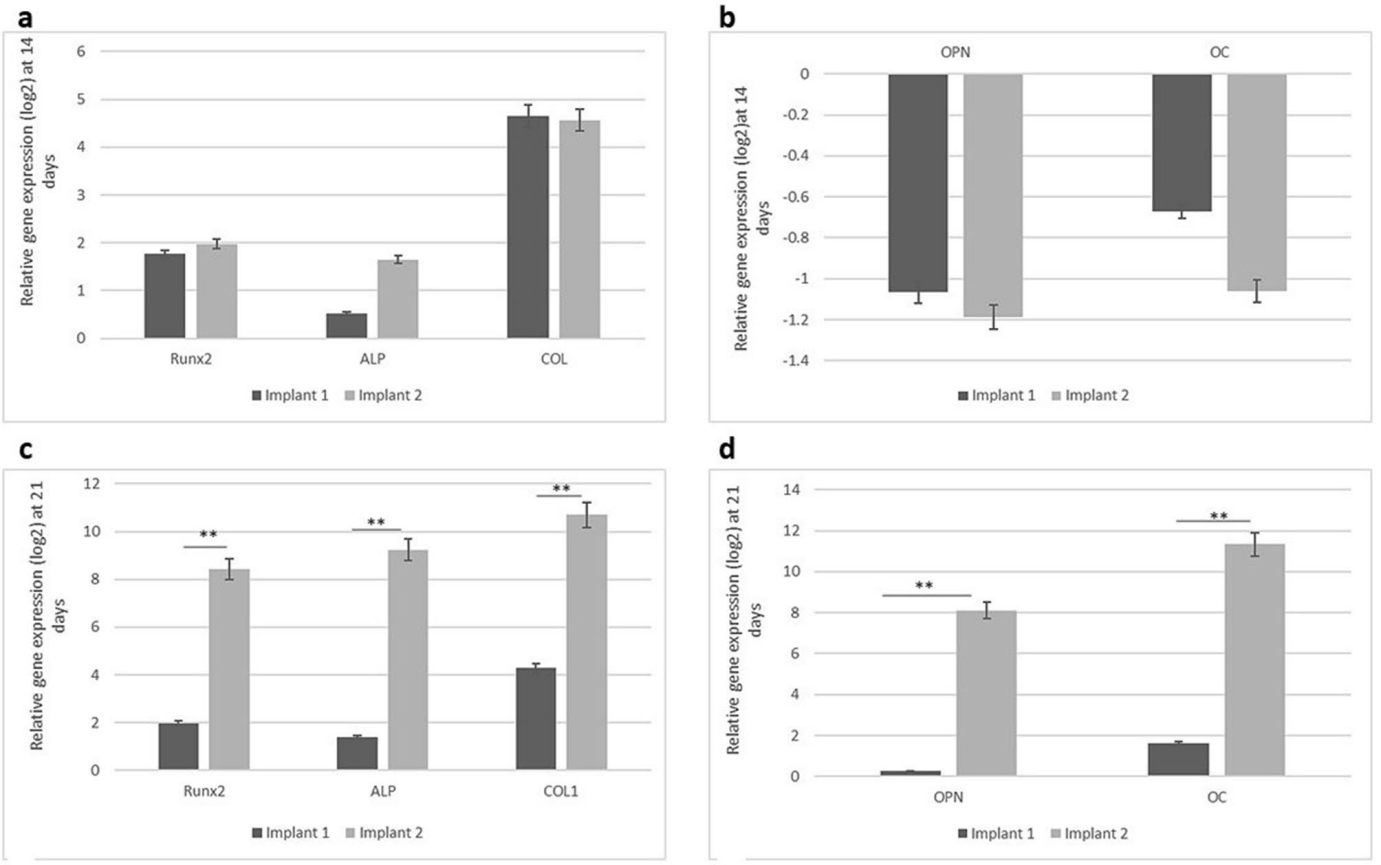
Quantitative real-time PCR analysis of the levels of mRNA for osteogenesis-related genes in hPDLSCs cultured on the investigated surfaces at 14 and 21 days of culture. (**a**) mRNA levels of early transcription factor genes for osteogenic differentiation (Runx2, COLI, and ALP) at 14 days of culture. (**b**) mRNA levels of osteocalcin (OC), and osteopontin (OPN) at 14 days of culture. (**c**) mRNA levels of early transcription factor genes for osteogenic differentiation (Runx2, COLI, and ALP) at 21 days of culture. (**d**) mRNA levels of osteocalcin (OC), and osteopontin (OPN) at 21 days of culture. Values were normalized for GAPDH. data are the mean ± SD of three independent experiments. ∗p < 0:05 between the two challenged implants. Laser surface treated (Implant 1) acid etched implant (Implant 2).

**Figure 9:**
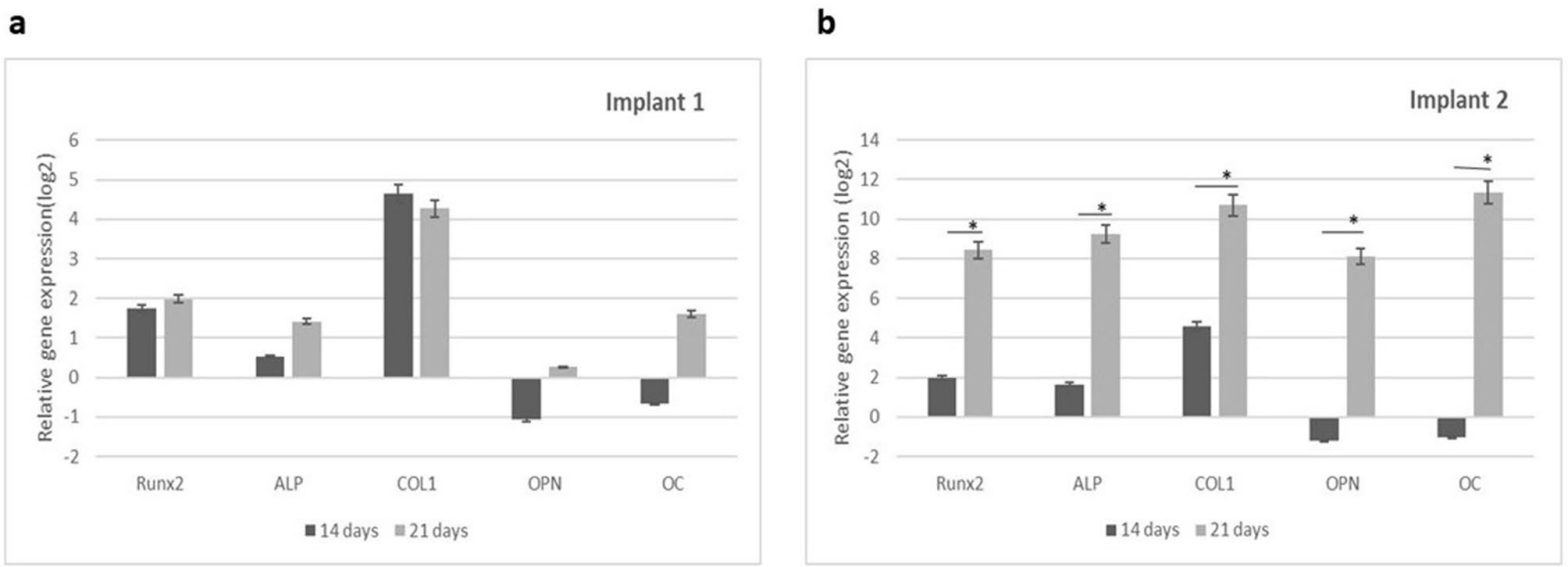
Relative gene expression of osteogenic markers at 14 and 21 days. (**a**) Laser surface treated (Implant 1) with relative increase in expression levels. (**b**) Acid etched implant (implant 2) with highly significance increase in levels of expression during osteogenic differentiation at 14 days and 21 days. ∗p < 0:05 with the two stages of differentiation time. Laser surface treated (Implant 1) acid etched implant (Implant 2).

The results of qRT-PCR analysis for certain master regulator genes of osteoblast differentiation, following 14 and 21 days of the two implants surfaces when challenged with osteogenic media and infrared diode laser irradiation are shown in (Fig. 8a–d). The gene expression of the early osteogenic differentiation markers: runt-related transcription factor 2 (Runx2), ALP and collagen type I (CoLI), and late osteogenic markers: osteocalcin (OC) and osteopontin (OPN) were assessed. At 14 days, early osteogenic markers, runt-related transcription factor-2 (RUNX2), type I collagen (COLI), alkaline phosphatase (ALP), were upregulated but without any statistically significant difference between the two challenged implants (Fig. 8a). While the late osteogenic markers, osteocalcin (OC), and Osteopontin (OPN) were downregulated in both challenged groups (Fig. 8b). At 21 days, hPDLSCs grown on acid etched implant surface displayed a statistically significant greater expression level of marker genes when compared with Laser surface treated implant for the early gene markers, RUNX2 (p = 0.003), COL1(p = 0.0008); and ALP (p = 0.0005) (Fig. 8c). In addition, mRNA expression of mature osteoblast phenotype genes, i.e., OC (p = 0.0007), and OPN (P = 0.002) were significantly upregulated (Fig. 8d). Interestingly, there was significance increase in the level of expression of the osteogenic markers in hPDLSCs seeded in acid etched implant upon increasing the differentiation time (Fig. 9b) while the cells that were seeded on laser surface treated implant showed relatively minimal increase in expression levels (Fig. 9a).

## Discussion

**Figure 10:**
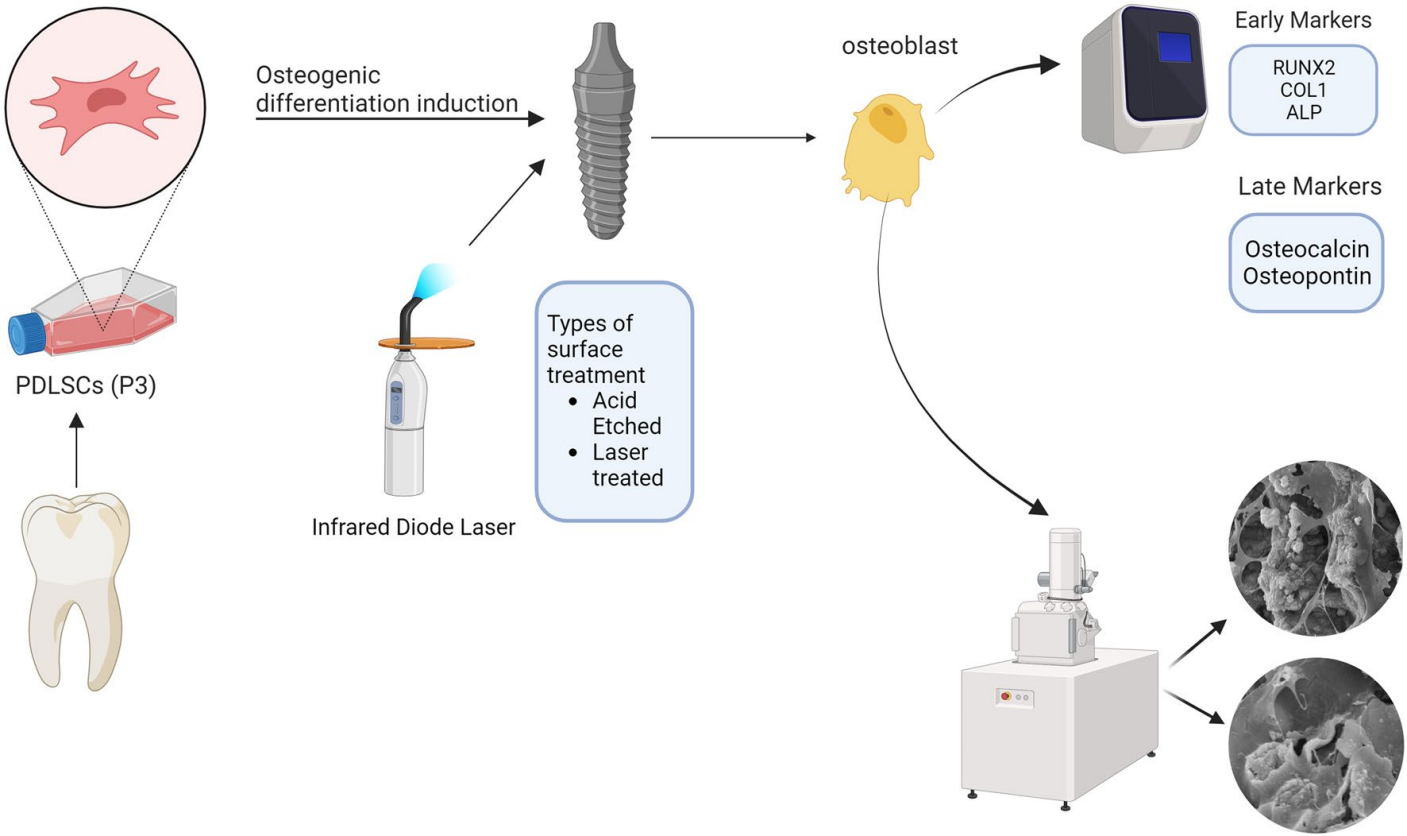
Schematic presentation of the experimental procedures.

The clinical success of any dental implant-based treatment depends mainly on the formation of new bone around the implant to achieve successful osseointegration. Thus, the primary objective of any treatment is to enhance and speed up bone formation in the vicinity of dental implants. Previous studies have documented the utilization of infrared diode lasers on dental implants to promote osteointegration^16,17,19^. Nevertheless, there is a lack of clinical and laboratory evidence investigating the efficacy of laser application in conjunction with periodontal ligament stem cells. The current investigation presented evidence that the application of infra-red diode laser to hPDLSCs in proximity to dental implants resulted in enhanced cellular proliferation and osteogenic differentiation, indicative of successful bone formation and integration with the implants, which is crucial for the success of dental implants (Fig. 10).

An effective method for achieving successful dental implants is to establish favourable conditions to recruit stem cells to the surface of the implants. Stem cells play a crucial role in this respect through establishing a regenerative microenvironment that promotes osteogenic differentiation and enhances tissue repair. Consequently, it is crucial to first employ an appropriate population of stem cells and to create favourable conditions surrounding the implant surface to induce osteogenic differentiation, thereby enhancing osteointegration^28^. This is why PDLSCs are considered good candidates for this specific purpose^29–33^.

We investigated the behavior of hPDLSCs seeded on two types of commonly used dental implants with different surface treatments in terms of attachment, proliferation, and osteogenic differentiation potential. Furthermore, we examined the impact of infrared diode laser (970 nm) at a power of 200 mW, fluence 4 J/cm^2^, which is readily accessible, cost-effective, and safe compared to other lasers, on hPDLSCs seeded on both types of implants.

Several techniques have been proposed to enhance cell attachment on implant surfaces, including as acid etching, sand blasting, and laser therapy^33^. All of which with the aim of creating surface roughness to facilitate cellular adhesion and to promote tissue formation on the implants’ surface. In the current study, we investigated two types of commercially available dental implants. The first type of implant investigated was the Laser Lok^®^ microchannels system. The implant exhibits uniformly aligned microstructures obtained through laser ablation. According to previous reports, this kind of textured surface results in enhanced stability and osseointegration^34,35^. The second type of implant investigated was the OXY K1^®^ dental implant. To improve cellular attachment, the surface of this implant is etched with acid plasma at low temperature to generate a uniform microporosity with reduced interpeak distance. The importance of the implant surface for promoting cellular attachment and osseointegration is widely acknowledged. Consequently, in our study, after seeding of hPDLSCs, both implant surfaces were exposed to infrared diode laser irradiation with the aim of enhancing cellular growth and attachment to the implant surface.

We initially examined the proliferative capacity of hPDLSCs on the surfaces of both implants using CCK-8 assay. Our results demonstrated a consistent pattern of increased growth of irradiated hPDLSCs on both types of implants, with an initially slow proliferation rate that gradually accelerated over time. The results were consistent with prior research and demonstrated that both implants were biocompatible and effectively promoted cell growth^13,15,20,21,24^.

Following proliferation investigations, hPDLSCs were induced to differentiate into osteogenic lineage via changing the culture media to osteogenic differentiation media coupled with infrared diode laser application periodically with every change of medium which was three times a week for 21 days. The osteogenic differentiation potential of the two studied implants were determined through assessment of gene expression of early osteogenic differentiation markers: runt-related transcription factor 2 (Runx2), ALP and collagen type I (CoLI), and late osteogenic markers: osteocalcin (OC) and osteopontin (OPN). The molecular analysis from our study demonstrated that the capacity of hPDLSCs to differentiate into osteogenic-like cells was substantially enhanced on acid etched implants compared to laser treated ones. This finding indicates that the application of infrared diode laser on hPDLSCs may have altered the surface of the implants to render it more suitable for cells to attach to.

Following a 14-day period of differentiation, during which infrared diode laser was consistently applied, early osteogenic markers were expressed while late osteogenic markers, were downregulated in hPDLSCs seeded on both types of implants. The results confirmed that hPDLSCs started differentiation with commitment to osteoprogenitor lineage. After 21 days, during which differentiation induction was combined with infrared diode laser irradiation, a statistically significant increase in the expression of all osteogenic markers was observed in hPDLSCs seeded on acid etched implants, as compared to those seeded on laser surface treated implants. This finding indicates that as the duration of differentiation increases, the expression of osteogenic markers in hPDLSCs seeded on acid etched implants considerably increases. To provide a comprehensive understanding of these findings, it is crucial to note that both OCN and OPN are non-collagenous proteins found in bones, and play a role in the process of matrix mineralization^36^. Phosphorylated glycoprotein OPN is believed to be present throughout the initial phases of bone formation, supporting the attachment of osteoblasts to the extracellular matrix, and actively participating in bone resorption^37,38^. Furthermore, OCN serves as the most precise indicator in the process of osteoblast maturation. These results suggest that the acid etched implant exhibited the highest ability to promote osteogenic differentiation in hPDLSCs.

Subsequently, we analyzed the cellular attachment of irradiated hPDLSCs on both implant surfaces using scanning electron microscopy (SEM). Our findings demonstrated that hPDLSCs exhibited robust proliferation and strong attachment to the surfaces of the implants. Moreover, hPDLSCs demonstrated a cellular sheet-like appearance after 14 days. By the end of differentiation induction, which occurred after 21 days, the cellular sheet appeared to have grown and migrated in order to attach to the structural topography of the implants. This pattern paralleled findings from prior research examining different implant types^20,24,36,37,39^. On the other hand, it appeared that hPDLSCs cultured on laser-treated implants attached to the microgrooves and laser-machined microchannels. This led to the cells presenting a channelled appearance, which is precisely what this design intended to promote horizontal cellular growth and attachment in a consistent manner as opposed to the random growth observed on the acid-etched implant.

Energy dispersive X-ray spectroscopy (EDX) was employed in conjunction with scanning electron microscopy (SEM) to analyse the mineral composition of the cells at both time points. SEM–EDX revealed the presence of calcium (Ca), Oxygen (O_2_), Phosphorus (P), Carbon (C) at days 14 and 21 whereas Fluorine (FL) was only detected after 14 days. We tried to find an explanation for this pattern of element analysis, and by searching related studies, we found that the amount of O and F detected usually corresponds to a higher cell adhesion and that increased F content corresponds to reduced cellular differentiation which explains why it was only present after 14 days and was not detected after 21 days where complete differentiation was achieved. It is worthy to mention, that both Ca and P detected were indicative of increased mineralisation. This finding is similar to the trends presented by EDX analysis in similar studies, whereby the surface treatment alters the chemical compositions^38^. Taken together, qRT-PCR results of osteogenic related markers expression and the surface chemistry characterization by SEM–EDX at two-point intervals, imply the compelling evidence that hPDLSCs cultured on both types of implants and subjected to infrared diode laser irradiation successfully differentiated towards osteogenic lineage.

In conclusion, the current study demonstrated that the use of an infrared diode laser (970 nm) with a power of 200 mW and a fluence of 4 J/cm^2^ enhanced the proliferation and attachment of hPDLSCs on dental implants' surfaces. In addition, these parameters improved the biological effectiveness and osteoinductive characteristics of dental implants, particularly acid etched implants. This study faced a few limitations. We only evaluated the impact of 970 nm infrared diode laser. It is advisable to conduct tests on alternative laser types. Furthermore, though the qrt-PCR markers that were evaluated indicates bone formation which is a crucial element of osseointegration it is advisable to further utilize the findings of the current investigation in an in-vivo experimental model to comprehensively assess the rate and quality of bone regeneration as well as the efficacy of osseointegration.

Finally, our results indicated that the combination of periodontal ligament stem cells and laser therapy on the implant surface can expedite bone formation and osseointegration. Moreover, the bone induced by periodontal ligament stem cells (PDLSCs) and enhanced by laser treatment is expected to have positive effects on the initial stability and movement pattern of dental implants. This may lead to significant improvements, including a reduction in marginal bone loss and an improvement in the recovery of bone quality. As a result, this approach will shorten the healing period after implantation and improve the long-term durability of the implant.

## Material and methods

**Figure 11:**
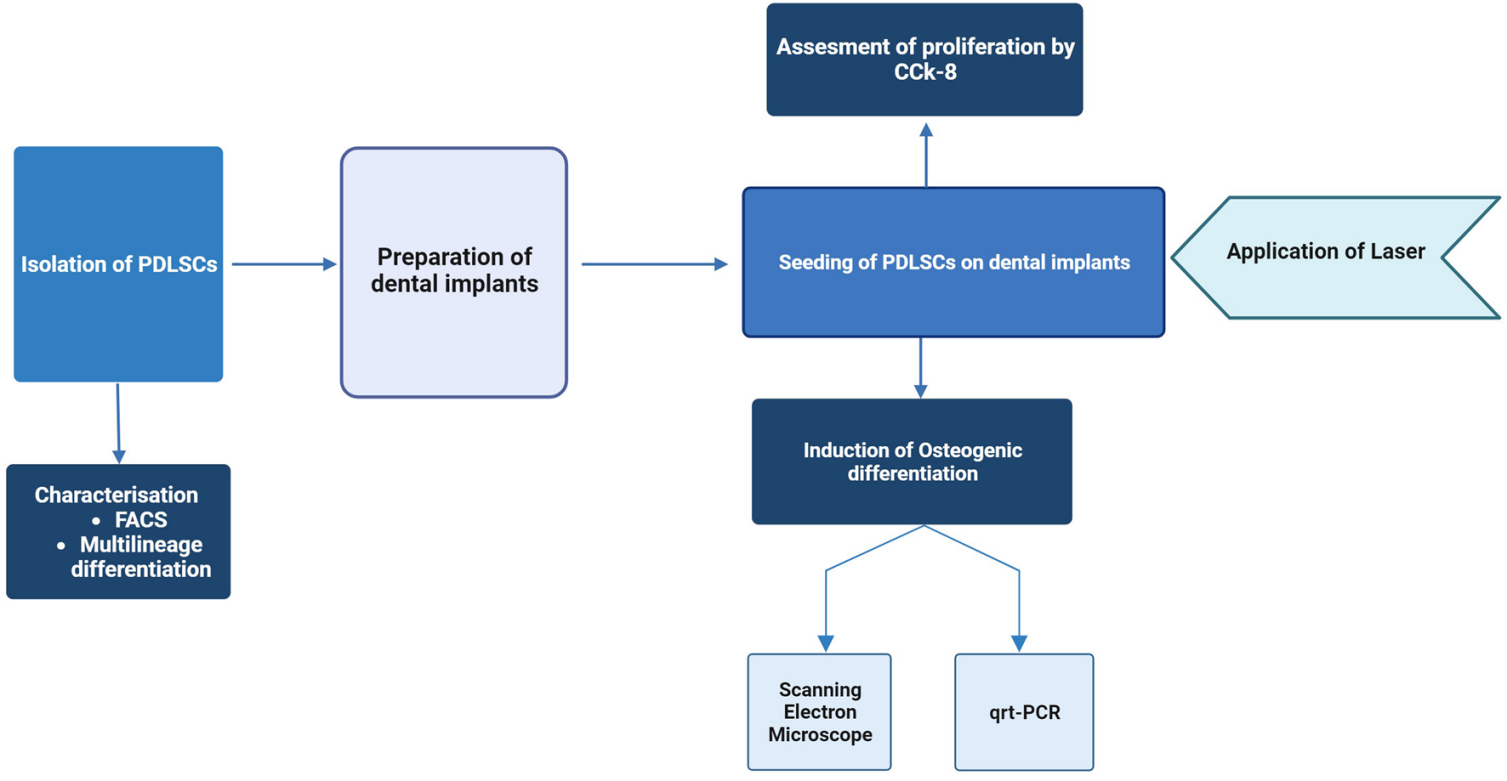
Flowchart outlining the methodology.

A schematic presentation of the experimental procedure is illustrated in Fig. 11.

### Isolation of human periodontal ligament stem cells (hPDLSCs)

Healthy human impacted third molars (n = 3) were placed in 50 ml sterile polypropylene tube containing Dulbecco modified Eagle’s medium (DMEM) supplemented with antibiotics (100 U/ml penicillin and 100 mg/ml streptomycin) immediately after extraction^7^. Periodontal ligament tissue was scraped off the surface of the roots by sterile lancet, rinsed five times by phosphate buffered solution (PBS) supplemented with penicillin and streptomycin. Periodontal ligament pieces were mixed and minced into tiny pieces, then digested in a solution of 2 mg/ml collagenase type I (Serva Electrophores, Germany) for 30 min at 37 °C in a water bath shaker. Single cell suspensions were seeded into culture plates with DMEM (Lonza), supplemented with 15% foetal bovine serum (Lonza), 10,000 µU/ml penicillin and 10,000 ng/ml streptomycin and then incubated in a humidified atmosphere of 5% CO_2_ at 37 °C. Cells were periodically monitored by phase contrast inverted microscopy and the culture medium was changed 3-times per week, the cells were sub-cultured when reached about 70% confluency by using 0.25% trypsin and 0.02% EDTA^31^.

### Flow cytometry analysis

To ensure that the cells in cultures are mesenchymal stem cells (MSCs), flow cytometry analysis was performed for MSCs specific markers (CD 73 and CD44) and hematopoietic marker (CD34)^40,41^. The PE conjugated CD 73 and CD 44 antibodies were purchased from R&D Systems (UK). Isotypes were used as controls. The cells were incubated with the antibody against each of the surface markers for 20 min at 25 °C followed by flow cytometry analysis using Beckman Coulter Elite XL, USA instrument.

### Dental implants preparation

In this study two types of implants were employed. The first type was surface etched with acid plasma at cold (OXY K1^®^, Biomec, Italy). The second type used was titanium laser micro-grooved implant (Laser-Lok^®^ microchannels, BioHorizons, Birmingham, Alabama). Both types of implants were cut longitudinally into two halves, sterilized, and placed in the center of six well culture plate with the flat cut side resting on the culture plate and the serrated side facing upward.

### Seeding of stem cells on dental implants

hPDLSCs of confluent third passages were re-suspended in DMEM and were seeded into six well plates having each type of implant placed at its center at a density of 3 × 10^4^ cells. Cells were cultured and left to proliferate on the implant surface for five days. During this time cells were checked closely via inverted microscope and cell proliferation was evaluated before switching the media to osteogenic induction media. Cells of passage 3 were characterized by flow cytometry for positive expression of mesenchymal stem cells markers (CD44 & CD73) and negative expression of hematopoietic marker (CD 34).

### Laser treatment of hPDLSCs seeded on the dental implants

hPDLSCs seeded on each of the implants were subjected to infrared diode laser (970 nm) with the following parameters: Power 200 mW, fluence 4 J/cm^2^, for 3 s: this dose has been reported to stimulate dental derived stem cells proliferation in vitro^42^. Uniform movements over the entire surface of the implant were applied. Control plates were not irradiated.

### Cell proliferation assay

On days 1, 3 and 5, cells were collected from each implant surface, rinsed with PBS, and transferred to 96-well plate. To each well, 450-μl of serum-free DMEM and 50-μl CCK-8 solution were added. Cells were incubated at 37 °C for 2 h. The optical density at 450 nm was measured using a spectrophotometer.

### Osteogenic differentiation

Osteogenic differentiation was induced through switching the culture media to osteogenic induction medium composed of DMEM, 10% FBS, 5 mM β-glycerol phosphate, 100 μM L- ascorbic acid 2-phosphate, 0.01 μM dexamethasone, 2 mM l-glutamine, 100 units/ml penicillin, 100 mg/ml streptomycin and 1.8 mM monopotassium phosphate for 3 weeks. The medium was changed twice per week. With each change of media laser was applied to the cells in the same manner described previously.

### Scanning electron microscope (SEM)

The study of cellular morphology and attachment to both types of implant surfaces after laser irradiation was evaluated using a Quanta 200 SEM (FEI, OR, USA) scanning electron microscopic at  1500× and  5000× magnifications. The implants were prepared for SEM evaluation as follows; initially, the implants were fixed in 2% paraformaldehyde solution and then subjected to successive dehydration in increasing concentrations of ethanol. Then, they were sputter-coated with a 50 nm layer of gold in order to prevent microscope beam alteration. Thereafter, the implants were placed in a vacuum container and SEM images were taken.

### Scanning electron microscopy and energy dispersive X-ray spectrometry analysis (SEM EDX)

 Before studying the elemental composition of the samples, the spectrometer was calibrated against Co at an operating accelerating voltage of 15 kV. X-ray spectral microanalysis was conducted at an accelerating voltage of 15 kV, a beam current of 7 × 10^−9^ A, and a working distance of 10 mm.

### Real-time polymerase chain reaction (PCR) analysis of adhesion- and osteogenesis-related gene expression

Relative gene expression in hPDLSCs grown on the two types of implants was evaluated during osteogenic differentiation induction and after exposure to infrared diode laser. Osteogenesis-related genes, including runt-related transcription factor-2 (RUNX2), osteoblast phenotype genes (type I collagen (COL), alkaline phosphatase (ALP), osteocalcin (OC), and Osteopontin (OPN) were measured after 14 and 21 days. hPDLSCs seeded on the two implants and maintained in 10% FBS basal medium without any differentiation factors were used as control groups. total cellular RNA was extracted by using RNeasy mini kit (Qiagen, Venlo, Netherlands). cDNA was synthesized from total cellular RNA using SuperScript III first-strand synthesis system (Invitrogen). Quantitative reverse transcription-polymerase chain reactions (qRT-PCR) reactions were performed using the corresponding primers. Glyceraldehyde-3-phosphate dehydrogenase (GAPDH) was used as a housekeeping reference gene in both experiments. Quantification was carried out on the PCR system (LC480, Roche) using the following cycle protocol: 1 cycle at 50 ◦C for 2 min, 1 cycle at 95 ◦C for 10 min, 40 cycles at 95 ◦C for 15 s, and 1 cycle at 60 ◦C for 1 min. The expression of each osteogenic gene was expressed as the CT (cycle threshold) value relative to that of the control (C-P). All experiments were repeated twice.

### Statistical analysis

All cell culture experiments were carried out in triplicates. Analysis of variance (ANOVA) followed by post hoc multiple comparison to determine differences within each group. A statistical significance was obtained for p < 0.05.

### Ethics approval

The authors are accountable for all aspects of the work in ensuring that questions related to the accuracy or integrity of any part of the work are appropriately investigated and resolved. All methods were performed in accordance with the guidelines and regulations of the Declaration of Helsinki. Informed consent has been obtained from all participants. The experimental protocol was reviewed by the Ethical Committee of the Medical Research of the National Research Centre, Egypt and granted approval under the number 12310112021.

## Data Availability

The datasets generated during and/or analyzed during the current study are available from the corresponding author on reasonable request.
